# Breast Neurotization in Implant-Based Breast Reconstruction: Results of a Split-Chest, Prospective, Randomized Controlled Trial

**DOI:** 10.1093/asjof/ojag138.020

**Published:** 2026-07-24

**Authors:** Kshipra Hemal, Carter J Boyd, Thomas J Sorenson, Samantha Lu, Brooke Miller, Nolan S Karp, Mihye Choi

**Affiliations:** Hansjörg Wyss Department of Plastic Surgery, NYU Langone, New York, New York; Hansjörg Wyss Department of Plastic Surgery, NYU Langone, New York, New York; Hansjörg Wyss Department of Plastic Surgery, NYU Langone, New York, New York; Hansjörg Wyss Department of Plastic Surgery, NYU Langone, New York, New York; Hansjörg Wyss Department of Plastic Surgery, NYU Langone, New York, New York; Hansjörg Wyss Department of Plastic Surgery, NYU Langone, New York, New York; Hansjörg Wyss Department of Plastic Surgery, NYU Langone, New York, New York

## Abstract

**Purpose:**

Reduced nipple and breast sensation negatively impact patient-reported outcomes following mastectomy. Recent reports suggest neurotization of the nipple-areolar complex (NAC) can help restore sensation in patients undergoing implant-based breast reconstruction (IBBR) following nipple-sparing mastectomy (NSM). Data from these interventions exists in small case series with no comparison group, making it difficult to draw definite conclusions. The goal of this study is to assess the effect of neurotization in IBBR following NSM on return of nipple and breast sensation using objective measures and study the patient’s perception of sensation and quality of life using standardized scales.

**Methods:**

This is a split-chest, randomized controlled trial designed to determine the effects of breast neurotization in IBBR following NSM. Patients undergoing bilateral IBBR following NSM were included. Preoperative and post-operative nipple and breast sensation was determined in both breasts using Semmes-Weinstein Monofilament Test (SWMT), measurements were taken by a provider (BM) who was blinded to randomization. Patients completed pre-and post-operative BREAST-Q questionnaires regarding breast sensation and breast symptoms. Patients were randomized to receive neurotization on one breast and not the other. Intraoperatively, the nipple assigned to be neurotized was connected to a donor intercostal nerve using standard nerve grafting techniques.

**Results:**

Six patients were included. Patients were on average 40 years old, white (83%), nonsmokers (100%), non-diabetic (100%), and had an average body mass index of 22 kg/m2 . Follow up at 12-months was available for four patients.

Preoperatively, median force required for sensation at the nipple, areola, and four regions of the breast was not significantly different between neurotized and non-neurotized breasts respectively (p=ns). At 1, 3, 6, and 12 months postoperatively, median force required for sensation increased in all regions and was not significantly different between neurotized and non-neurotized breasts at each time point (Figure 1).

Patient-reported breast sensation and symptoms were best in the pre-operative period, declined after surgery, and did not return to baseline at the 12-month mark; no significant differences between neurotized and non-neurotized breasts were observed at any timepoint. Lastly, patients and a blinded provider were asked to guess which breast was neurotized, and did so correctly 69% and 63% of the time.

**Conclusions:**

In a split-chest, randomized control trial of breast neurotization, we have identified no significant difference in objective metrics of nipple and breast sensation or patient-reported outcomes at the 12-month mark. Ultimately, the challenges of performing a Level I study have limited the sample size of this cohort, though with ongoing data collection we hope to provide further clarity to the efficacy and impact of neurotization following NSM.

